# IL-10 produced by macrophages regulates epithelial integrity in the small intestine

**DOI:** 10.1038/s41598-018-38125-x

**Published:** 2019-02-04

**Authors:** Tina L. Morhardt, Atsushi Hayashi, Takanori Ochi, Miguel Quirós, Sho Kitamoto, Hiroko Nagao-Kitamoto, Peter Kuffa, Koji Atarashi, Kenya Honda, John Y. Kao, Asma Nusrat, Nobuhiko Kamada

**Affiliations:** 1 Division of Gastroenterology, Department of Internal Medicine, University of Michigan, Ann Arbor, MI USA; 2 Division of Pediatric Gastroenterology, Department of Pediatrics, University of Michigan, Ann Arbor, MI USA; 3 Department of Pathology, University of Michigan, Ann Arbor, MI USA; 4 Research Laboratory, Miyarisan Pharmaceutical Co., Ltd, Tokyo, 114-0016 Japan; 5 Department of Pediatric General and Urogenital Surgery, Juntendo University School of Medicine, Tokyo, Japan; 6 Department of Microbiology and Immunology, Keio University School of Medicine, Tokyo, Japan

## Abstract

Macrophages (Mϕs) are known to be major producers of the anti-inflammatory cytokine interleukin-10 (IL-10) in the intestine, thus playing an important role in maintaining gastrointestinal homeostasis. Mϕs that reside in the small intestine (SI) have been previously shown to be regulated by dietary antigens, while colonic Mϕs are regulated by the microbiota. However, the role which resident Mϕs play in SI homeostasis has not yet been fully elucidated. Here, we show that SI Mϕs regulate the integrity of the epithelial barrier via secretion of IL-10. We used an animal model of non-steroidal anti-inflammatory drug (NSAID)-induced SI epithelial injury to show that IL-10 is mainly produced by MHCII^+^ CD64^+^ Ly6C^low^ Mϕs early in injury and that it is involved in the restoration of the epithelial barrier. We found that a lack of IL-10, particularly its secretion by Mϕs, compromised the recovery of SI epithelial barrier. IL-10 production by MHCII^+^ CD64^+^ Ly6C^low^ Mϕs in the SI is not regulated by the gut microbiota, hence depletion of the microbiota did not influence epithelial regeneration in the SI. Collectively, these results highlight the critical role IL-10-producing Mϕs play in recovery from intestinal epithelial injury induced by NSAID.

## Introduction

The intestine contains the largest pool of macrophages (Mϕs) in the body. They produce the anti-inflammatory cytokine interleukin (IL)-10, which is essential for the maintenance of mucosal homeostasis^1–3^. Intestinal Mϕs differentiate from bone marrow-derived Ly6C^hi^CCR2^+^ monocytes and are constantly replenished by circulating Ly6C^hi^CCR2^+^ monocytes at steady-state^4^. Once in the gut, the Ly6C^hi^CCR2^+^ monocytes down-regulate the expression of Ly6C and CCR2 under homeostatic conditions^4,5^. Subsequently, they give rise to tissue resident Mϕs, and express MHC-II and CX_3_CR1^4^. Once homeostasis is perturbed by inflammation, Ly6C^hi^CCR2^+^ monocytes and their CX_3_CR1^int^ derivatives, both of which display enhanced pro-inflammatory characteristics, accumulate in large numbers in the intestine^6^. Thus, intestinal Mϕs play a regulatory role in the gastrointestinal tract at steady state as well as during inflammation. IL-10 was first found to have immuno-regulatory effects in the intestine upon the discovery of spontaneous enterocolitis in IL-10-deficient mice^7^. In humans, IL-10 and IL-10 receptor polymorphisms have been found to be monogenic causes of very early onset inflammatory bowel disease (VEO-IBD)^8^. Medically and surgically refractory disease manifests itself in these patients who ultimately require a stem cell transplant^8,9^. Overall, IL-10 is an important anti-inflammatory cytokine involved in the regulation of intestinal immunity.

Accumulating evidence indicates that the gut microbiota plays a crucial role in the regulation of IL-10-producing Mϕs in the colon^4,10,11^. Since colonic Mϕs and the IL-10 they produce are critical for epithelial regeneration in the colon^12,13^, depletion of the microbiota significantly impairs recovery from colonic epithelial injury. While the gut microbiota is clearly important for colonic homeostasis, its contribution to homeostasis in the small intestine (SI) is likely limited. For example, the generation and maintenance of regulatory T cells in the SI have been recently shown to be controlled by dietary antigens. This stands in contrast to the colon where these processes are microbiota-dependent^14,15^. Consistent with this, we have previously reported that IL-10-producing Mϕs in the SI are regulated by dietary factors^16^. The IL-10-producing Mϕs in the SI are less abundant and consequently produce less IL-10 in a total parenteral nutrition mouse model in which mice do not experience enteral stimulation by dietary antigens^16^. Notably, this animal model is associated with gut translocation and impairments of the intestinal barrier^16^. As such, the dietary antigen-dependent population of Mϕs in the SI may also be involved in intestinal homeostasis. Specifically, we hypothesized that IL-10-producing Mϕs might contribute to the regulation of epithelial barrier integrity in the SI.

In order to study the role Mϕs play in maintaining homeostasis in the SI, we employed the non-steroidal anti-inflammatory drug (NSAID)-induced epithelial injury model. Administration of NSAIDs, such as indomethacin (IND), is well known to induce epithelial injuries in the upper gastrointestinal tract, including the SI, a serious clinical event in both human and mouse applications^17^. The mechanism and appropriate treatment of NSAID-induced SI injury have not been completely elucidated. Using this mouse model, we determined that IL-10-producing MHCII^+^ CD64^+^ Ly6C^low^ Mϕs play an essential role in the recovery from acute SI injury.

## Results

### Indomethacin induces epithelial injury in the SI

**Figure 1:**
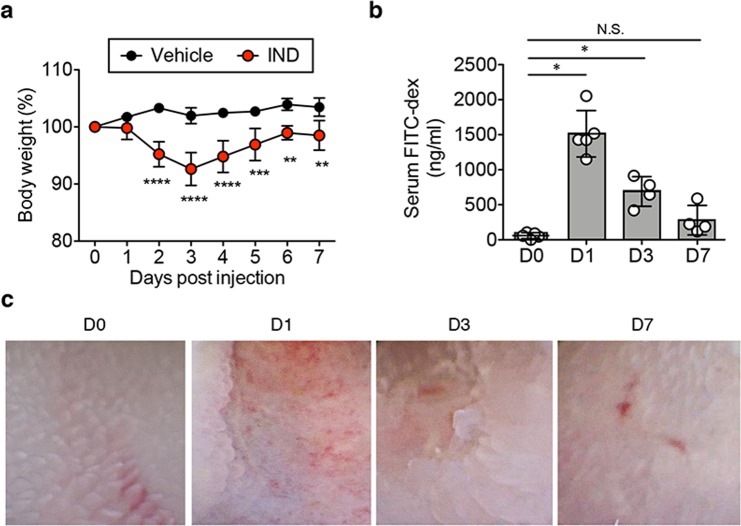
Indomethacin induces SI epithelial injury. (**a**) WT mice were subcutaneously injected with indomethacin (IND) (15 mg kg^−1^ body weight) or vehicle alone (100 μl/mouse). Body weight loss from initial body weight is shown. Data are given as mean ± s.d. (Control; N = 3, IND; N = 8). *P < 0.05 by 2-Way ANOVA followed by Bonferroni post-hoc test. (**b**) Fluorescein-isothiocyanate (FITC)-labeled dextran was administered by oral gavage. Leakage of FITC-dextran from the gut was measured (in serum) one hour post administration. Assays were performed on indicated days post IND injection. Data are given as mean ± s.d. (N = 5). Dots indicate individual mice. N.S.; not significant, *P < 0.05 by Dunnett test. (**c**) SI endoscopy was performed on indicated days post IND injection. Representative endoscopic images are shown.

**Figure 2:**
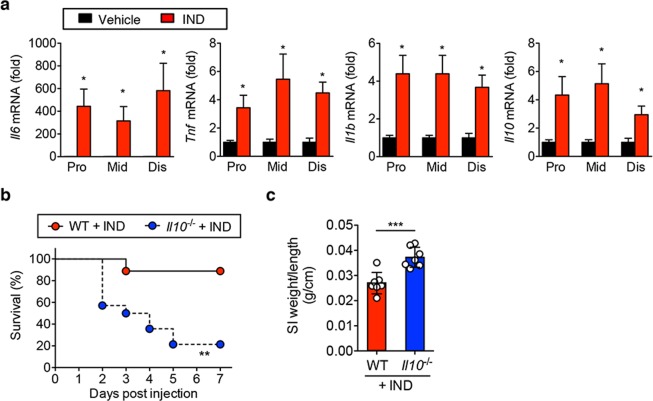
IL-10 is required for recovery from SI injury. (**a**) WT mice were subcutaneously injected with indomethacin (IND) (15 mg kg^−1^ body weight) or vehicle alone (100 μl/mouse). 24 hours after IND injection, proximal, middle, and distal regions of SI were harvested and the expression of pro- and anti-inflammatory cytokines was assessed. Data are given as mean ± s.d. (N = 8). *P < 0.05 by Student’s *t*-test (2-tailed). (**b**) WT and *Il10*^−*/*−^ mice were subcutaneously injected with IND (15 mg kg^−1^ body weight). Mouse mortality is shown (WT; N = 9, *Il10*^−*/*−^; N = 13). **P < 0.01 by Log-rank test. (**c**) Mice were sacrificed one day post IND injection. SI tissue was harvested and the severity of SI inflammation (SI weight per length) was evaluated. Dots indicate individual mice. Bars indicate mean (N = 7). ***P < 0.0001 by Student’s *t*-test (2-tailed).

A single dose of IND (15 mg/kg body weight) was adequate to induce SI epithelial injury in C57BL/6 wild-type (WT) mice. Maximum body weight loss was observed on day 3 and by day 7 the animals largely managed to regain their initial weight (Fig. 1a). Body weight loss corresponded with increased intestinal permeability, as quantified by a fluorescein isothiocyanate (FITC) dextran assay as well as endoscopic findings of ulceration and hemorrhage (Fig. 1b,c). Thus, IND can cause recoverable SI injury. To clarify the immune mechanism underlying the recovery from SI injury, we next assessed gene expression changes in the SI mucosa. Mucosal tissue samples were obtained from there different regions of the SI (proximal, middle and distal SI) during the early phase of SI injury (24 hours post IND injection). mRNA levels of pro-inflammatory cytokines, such as tumor necrosis factor-alpha (TNF-α), IL-6, and IL-1β, were significantly increased in all regions of the SI mucosa following IND injection (WT + IND) (Fig. 2a). Notably, the expression of anti-inflammatory cytokine IL-10 was likewise elevated in the SI mucosa during the early phase of SI injury (Fig. 2a). These results suggest that IL-10 may play a compensatory role in the early phase of injury, paving the way to recovery from epithelial damage and body weight loss.

### Deficiency of IL-10 exacerbates indomethacin-induced SI injuries

Next, we examined the role of IL-10 in the recovery from IND-induced SI injuries using IL-10-deficient (*Il10*^−/−^) mice. Compared to WT mice, *Il10*^−/−^ mice showed impaired recovery from SI injury, thereby resulting in higher mortality (Fig. 2b). The SI weight-to-length ratio was significantly increased in IND-treated *Il10*^−/−^ mice, indicating that *Il10*^−/−^ mice develop more severe intestinal inflammation as a result of IND treatment (Fig. 2c). These results suggest that in the absence of IL-10, SI epithelial tissue repair does not occur, in part due to unhindered effects of pro-inflammatory cytokines.

### IL-10 is primarily produced by macrophages during the acute phase of injury

**Figure 3:**
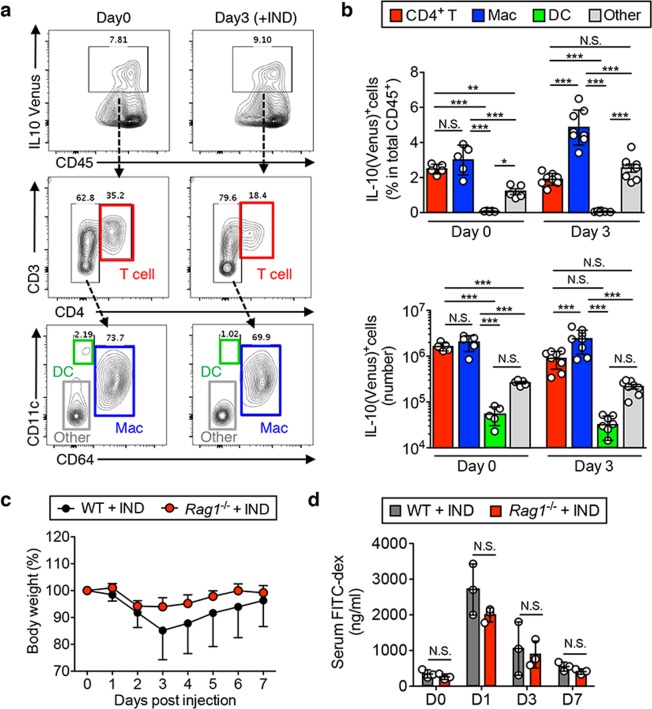
IL-10 production is primarily from macrophages during the acute phase of injury. (**a**,**b**) *IL-10*^Venus^ reporter mice were subcutaneously injected with indomethacin (15 mg kg^−1^ body weight) or vehicle alone (100 μl/mouse). Three days post IND injection, small intestinal samples were harvested and used to isolate SI LPMCs, which were subsequently analyzed by flow cytometry. (**a**) Representative FACS plots (day 0 and day 3) are shown. (**b**) Populations of IL-10-producing (Venus^+^) CD4^+^CD3^+^ T cells (T), CD64^+^ macrophages (Mac) and CD11c^+^CD64^−^ dendritic cells (DC) shown as percentages of total CD45^+^ cells (top) and depicted in absolute numbers (bottom). Data are given as mean ± s.d. (Day 0; N = 5, Day 3; N = 8). Dots indicate individual mice. N.S.; not significant, *P < 0.05 by 1-Way ANOVA followed by Bonferroni post-hoc test. (**c**) WT and *Rag1*^−/−^ mice were treated with IND. Changes in body weight were monitored for seven days post IND injection. Data are given as mean ± s.e.m. (WT; N = 7, *Rag1*^−/−^; N = 8). (**d**) FITC-labeled dextran was administered by oral gavage. Leakage of FITC-dextran from the gut was measured (in serum) one hour post administration. Assays were performed on indicated days post IND injection. Data are given as mean ± s.d. (N = 3). Dots indicate individual mice. N.S.; not significant by Student’s *t*-test (2-tailed).

We next examined the source of IL-10 *in vivo* by using IL-10 reporter mice (IL-10^Venus^ mice^15,18^). Based on the IND kinetics data (Fig. 1a), body weight loss peaked on day 3 post injection and then gradually returned to normal levels. In order to focus on IL-10 responses during this phase, we analyzed mice on day 3 post IND injection. Lamina propria mononuclear cells (LPMCs) were isolated from the SI of IND-treated IL-10^Venus^ mice on day 0 and day 3 following IND injection. IL-10-producing cells (Venus^+^) found in the total population of CD45^+^ SI LPMCs were analyzed by flow cytometry. We first gated on IL-10(Venus)^+^ cells within total CD45^+^ leucocytes (Fig. 3a top panel), and then further characterized by staining T cells and myeloid cells (Fig. 3a middle and bottom panels). Within the IL-10(Venus)^+^ CD45^+^ cells, helper T cell populations were gated as CD3^+^ CD4^+^ T cells (Fig. 3a middle panel). Non-T cell populations were further distinguished into Mϕs (CD64^+^), dendritic cells (DCs; CD64^−^CD11c^+^) and other cells (CD64^−^CD11c^−^) (Fig. 3a bottom panel). At steady-state, CD4^+^ T cells and Mϕs were the major producers of IL-10 in the SI mucosa (day 0 IND) (Fig. 3b). During the acute phase of SI inflammation (day 3 IND), CD64^+^ Mϕs became the predominant source of IL-10 (Fig. 3b). Although the abundance of IL-10-producing CD3^+^ CD4^+^ T cells was lower than that of Mϕs during SI injury, it is possible that IL-10 produced by T cells also contributed to recovery. T and B cell-deficient *Rag1*^−/−^ mice were used to exclude the involvement of T cell-derived IL-10 in this injury model (Fig. 3c). *Rag1*^−/−^ mice also experienced maximum body weight loss on day 3 following IND injection and then recovered normally (Fig. 3c). Consistently, intestinal permeability was increased on day 1 following IND administration but was restored by day 3 and day 7 in both WT and *Rag1*^−/−^ mice (Fig. 3d). These data indicate that Mϕs are the major source of IL-10 during SI inflammation and play a predominant role in the recovery from SI injury.

### MHC-II^+^ CD64^+^ Ly6C^low^ macrophages are responsible for IL-10 production

**Figure 4:**
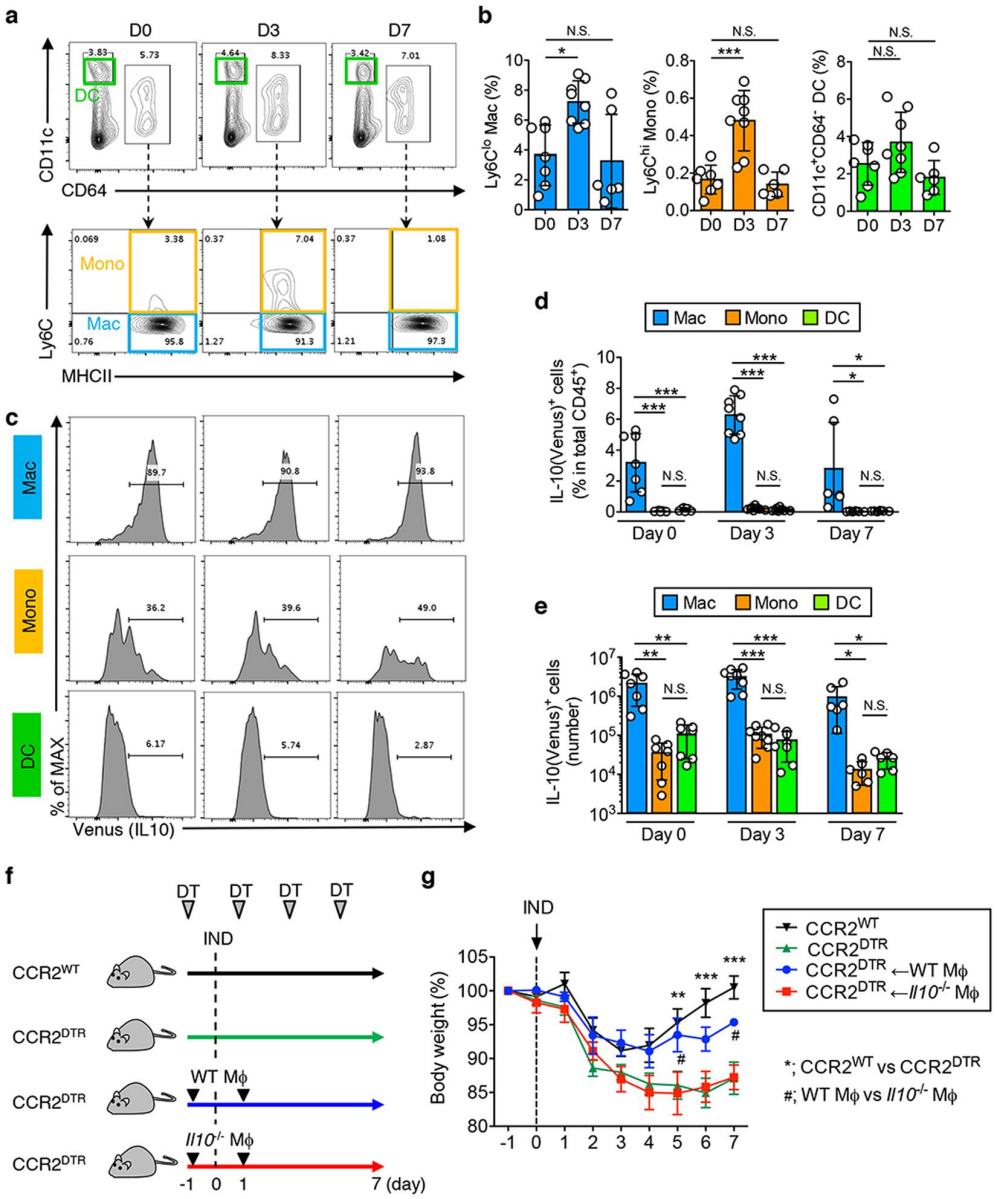
Monocyte-derived macrophages contribute to recovery from SI epithelial injury via secretion of IL-10. (**a**–**e**) *IL-10*^Venus^ reporter mice were subcutaneously injected with indomethacin (15 mg kg^−1^ body weight) or vehicle alone (100 μl/mouse). On day 0, 3 and 7 post IND injection, SI tissues were harvested and used to isolate LPMCs, which were subsequently analyzed by flow cytometry. (**a**) Representative FACS plots of CD45-gated SI LPMCs are shown (**b**) Percentages of newly recruited CD64^+^Ly6C^high^MHC-II^+^ monocytes, CD64^+^Ly6C^low^MHC-II^+^ resident/terminally differentiated Mϕs and CD11c^+^CD64^−^ DCs within total CD45^+^ cells are shown. Data are given as mean ± s.d. (Day 0; N = 7, Day 3; N = 8, Day 7; N = 6). Dots indicate individual mice. N.S.; not significant, *P < 0.05, ***P < 0.001 by 1-Way ANOVA followed by Dunnett post-hoc test (vs Day 0). (**c**) Representative histograms showing IL-10 (Venus) expression in newly recruited CD64^+^Ly6C^high^MHC-II^+^ monocytes, resident/terminally differentiated CD64^+^Ly6C^low^MHC-II^+^ Mϕs and CD11c^+^CD64^−^ DCs. (**d**,**e**) The percentage (**d**) and the absolute number (**e**) of newly recruited IL-10-producing (Venus^+^) CD64^+^Ly6C^high^MHC-II^+^ monocytes, resident/terminally differentiated CD64^+^Ly6C^low^MHC-II^+^ Mϕs, and CD11c^+^CD64^−^ DCs. Data are given as mean ± s.d. (Day 0; N = 7, Day 3; N = 8, Day 7; N = 6). Dots indicate individual mice. N.S.; not significant, *P < 0.05, **P < 0.01, ***P < 0.001 by 1-Way ANOVA followed by Bonferroni post-hoc test. (**f**) *CCR2*^DTR^ mice and littermate *CCR2*^*WT*^ mice were subcutaneously injected with IND (15 mg kg^−1^ body weight) or vehicle (100 μl/mouse). Diphtheria toxin (DT; 10 ng g^−1^ body weight) was i.p. injected every other day starting on day −1. CD11b^+^ Mϕs were isolated from the SI of WT or *Il10*^−/−^ mice and then adoptively transferred (2 × 10^6^ cells) into Mϕ-depleted mice (*CCR2*^*DTR*^ + DT injection). (**g**) Body weight loss relative to initial body weight is shown. Data are given as mean ± s.e.m. (CCR2^WT^; N = 4, CCR2^DTR^; N = 6, CCR2^DTR^ + WT Mϕ; N = 4, CCR2^DTR^ + *Il10*^−/−^ Mϕ; N = 6). *^,#^*P < *0.05, **P < 0.01, ***P < 0.001 by 2-Way ANOVA followed by Bonferroni post-hoc test. *CCR2^WT^ vs CCR2^DTR^. #CCR2^DTR^ + WT Mϕ vs CCR2^DTR^ + *Il10*^−/−^ Mϕ.

Next, we further characterized the IL-10-producing cell population during IND-induced SI injury. It is well-documented that intestinal inflammation promotes the infiltration of Ly6C^hi^ CCR2^+^ monocytes from peripheral blood into the lamina propria. Recruited Ly6C^hi^ monocytes then give rise to Mϕs locally. We sought to identify whether the newly recruited Ly6C^hi^ monocytes or resident/terminally differentiated monocyte-derived Mϕs are the major producers of IL-10 during SI injury. To this end, we isolated SI LPMCs from IL-10^Venus^ mice on day 0, day 3 and day 7 following IND injection. CD64^+^ monocyte/Mϕ lineage cells were then further separated into Ly6C^hi^ MHC-II^−/+^ newly recruited monocytes and Ly6C^lo^ MHC-II^+^ resident/terminally-differentiated Mϕs (Fig. 4a). CD11c^+^ CD64^−^ DCs were also analyzed as a control subset. At steady-state (day 0), a limited number of Ly6C^hi^ MHC-II^−/+^ monocytes were present in the SI LPMCs (Fig. 4a,b). At the peak of SI inflammation (day 3), a significant number of Ly6C^hi^ MHC-II^−/+^ monocytes were recruited into the SI mucosa, but these cells disappeared as SI inflammation resolved (day 7) (Fig. 4a,b). We next analyzed IL-10 expression in each subset. Although Ly6C^hi^ MHC-II^−/+^ monocytes produced a notable amount of IL-10, Ly6C^lo^ MHC-II^+^ resident/terminally-differentiated Mϕs remained the predominant sources of IL-10, even during inflammation (Fig. 4c,d,e). CD11c^+^ CD64^−^ DCs were a minor source of IL-10, both at steady-state and the inflamed SI mucosa (Fig. 4c,d,e). To address the role IL-10-producing Mϕs serve in the recovery from SI injury, we next attempted to deplete these cells. To this end, we used *CCR2*^DTR^ mice, as the CD64^+^ Ly6C^lo^ MHC-II^+^ resident/terminally-differentiated Mϕs arise from CCR2^+^ monocytes^4^ (Fig. 4f). Continual depletion of *CCR2*^+^ monocyte-derived Mϕs during IND-induced injury demonstrated a decreased ability to recover from epithelial damage as evidenced by the inability to return to initial body weight (Fig. 4g). In order to confirm that the IL-10-mediated recovery is Mϕ-dependent, we depleted Mϕs from *CCR2*^DTR^ mice and adoptively transferred SI Mϕs isolated either from WT or *Il10*^−*/*−^ mice (Fig. 4f). The animals that received WT intestinal Mϕs managed to regain most of their initial weight, indicating recovery from intestinal injury (Fig. 4g). On the other hand, the adoptive transfer of *Il10*^−*/*−^ intestinal macrophages resulted in a more significant weight loss and an inability to recover from intestinal injury, similar to mice lacking intestinal Mϕs altogether (Fig. 4g).

### The gut microbiota is not required for epithelial regeneration in the SI

**Figure 5:**
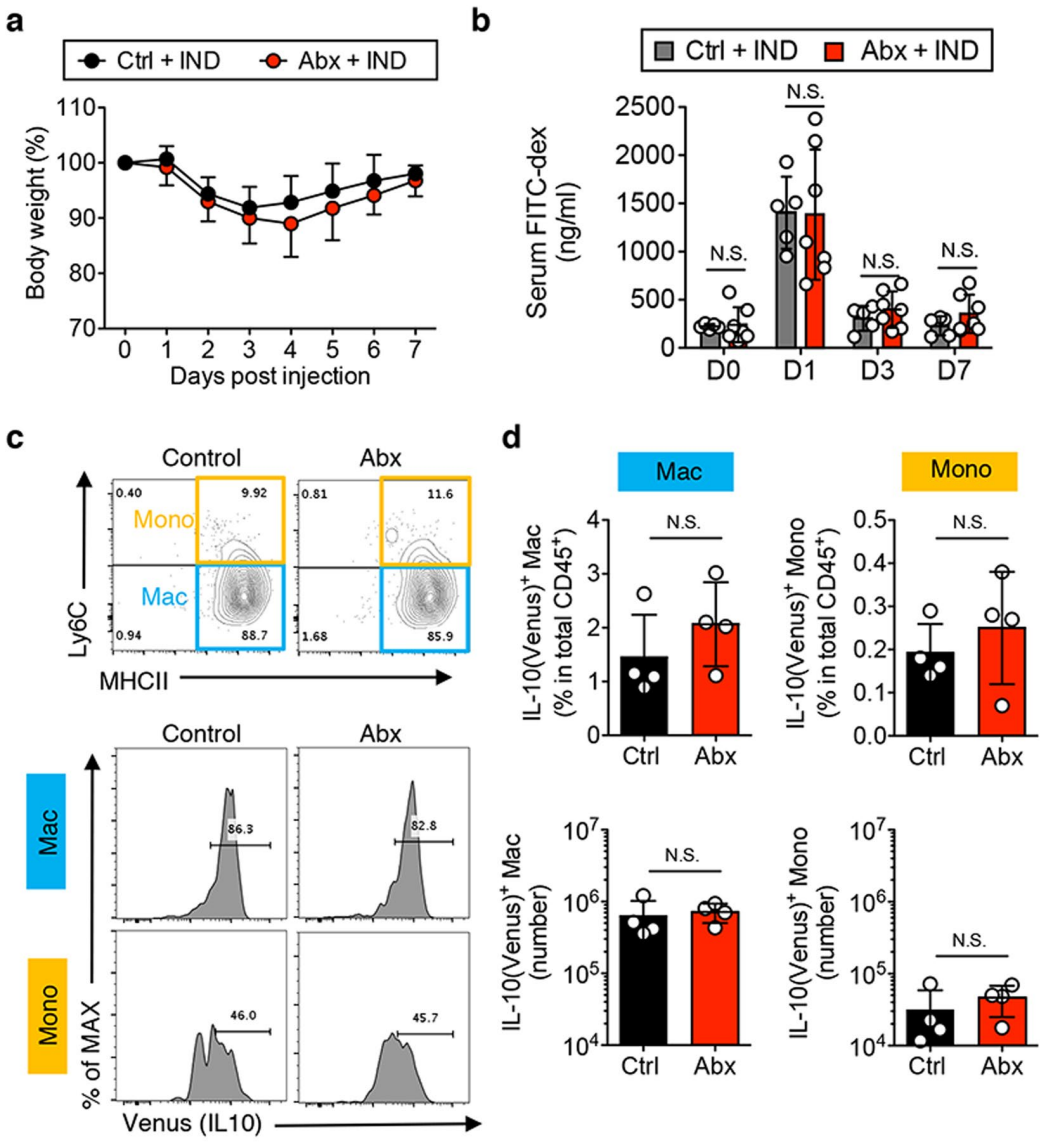
The gut microbiota is not required for recovery from SI injury. (**a**,**b**) SPF C57BL/6 mice were given a cocktail of antibiotic (Abx; vancomycin, neomycin, metronidazole and ampicillin) via oral gavage before (day −1) and after (day 1 and 3) IND injection. (**a**) Body weight change post IND injection. Data are given as mean ± s.e.m. (Control; N = 5, Abx; N = 7). (**b**) FITC-labeled dextran was administered by oral gavage. Leakage of FITC-dextran from the gut was measured (conc. in serum) one hour post administration. Assays were performed on indicated day post IND injection. Data are given as mean ± s.d. (Control; N = 5, Abx; N = 7). Dots indicate individual mice. N.S.; not significant by Student’s *t*-test (2-tailed). (**c**,**d**) *IL-10*^Venus^ reporter mice were given a cocktail of Abx via oral gavage before (day −1) IND injection. One day post IND injection, SI samples were harvested and used to isolate SI LPMCs. Cell populations found in LPMCs were analyzed by flow cytometry. (**c**) Representative FACS plots of Ly6C and MHC-II expression in CD45^+^CD64^+^ cells (top) and IL-10 (venus) expression in CD45^+^CD64^+^MHC-II^+^Ly6C^lo^ macrophages (Mac) and CD45^+^CD64^+^MHC-II^+^Ly6C^hi^ monocytes (Mono) (bottom) are shown. (**d**) The percentages of total CD45^+^ cells (top) and the absolute numbers (bottom) of IL-10-producing (Venus^+^) CD45^+^CD64^+^MHC-II^+^Ly6C^lo^ macrophages (Mac) and CD45^+^CD64^+^MHC-II^+^Ly6C^hi^ monocytes (Mono) are shown. Data are given as mean ± s.d. (N = 4). Dots indicate individual mice. N.S.; not significant by Student’s *t*-test (2-tailed).

Colonic Mϕs and IL-10 production by these cells are known to be regulated by the gut microbiota and its metabolites^4,10,11,19^. Hence, depletion of the gut microbiota significantly impairs epithelial recovery in the colon, in part due to insufficient activation of colonic Mϕs^12,20^. In contrast, we have previously demonstrated that IL-10-producing Mϕs in the SI are not regulated by the gut microbiota^16^. Thus, we thought it likely that Mϕ-mediated epithelial recovery in the SI is independent of the gut microbiota. To address this hypothesis, we depleted the gut microbiota by treating mice with a cocktail of four antibiotics (Abx; vancomycin, neomycin, metronidazole, and ampicillin) prior and during IND treatment. When compared to Abx-negative control mice, Abx-treated mice experienced similar body weight loss and recovery (Fig. 5a). Correlating with this observation, there was no significant difference in intestinal permeability during intestinal injury or recovery between control and Abx-treated mice (Fig. 5b). Consistently, the number of Mϕs and IL-10 production in the SI LPMCs were not affected by Abx-treatment (Fig. 5c,d). These results indicate that the gut resident microbiota does not regulate IL-10-producing Mϕs in the SI and the absence of the microbiota does not influence recovery from SI epithelial injury.

## Discussion

A single layer of epithelial cells lines the gastrointestinal tract. It separates the luminal contents, such as commensal bacteria and dietary antigens, from the immune cells in the lamina propria. The intestinal barrier can be disrupted by mechanical injuries, toxic agents, and drugs. In healthy individuals, disruption of the barrier is typically a transient event. Epithelial repair occurs rapidly following an injury, thus restoring barrier integrity. However, genetic predispositions and/or abnormal immune responses impair the re-epithelialization machinery. As a result, chronic exposure to the luminal antigens, such as commensal microbes, promotes excessive activation of immune cells in the lamina propria, thereby causing chronic intestinal inflammation. Thus, epithelial injury repair is a key determinant in balancing intestinal homeostasis and diseases, such as IBD.

In this study, we have shown that Mϕs contribute to recovery from SI injury in an IL-10-dependent manner. IL-10 is constitutively produced by Mϕs at steady-state as well as during inflammation, and the intestine contains the largest pool of IL-10-producing Mϕs in the body^2^. Using an animal model of drug (IND)-induced SI injury, we found that a significant number of Ly6C^hi^ monocytes are recruited to the SI mucosa. Since depletion of CCR2^+^ cells, a receptor abundantly expressed on Ly6C^hi^ monocytes, impairs recovery from IND-induced epithelial injury, recruitment of Ly6C^hi^ monocytes is crucial for re-epithelialization in the SI. However, Ly6C^hi^ monocytes are not the major source of IL-10 during the recovery phase. Thus, it is likely that the recruited Ly6C^hi^ monocytes do not directly promote epithelial repair. It has been reported that Ly6C^hi^CCR2^+^ monocytes down-regulate the expression of Ly6C and CCR2 in the gut^4,5^. Subsequently, they give rise to tissue resident Mϕs and express MHC-II and CX_3_CR1^4^. Thus, it is plausible that the recruited Ly6C^hi^ monocytes undergo terminal differentiation to become IL-10-producing MHCII^+^Ly6C^low^CD64^+^ Mϕs during SI injury and that these cells ultimately drive re-epithelialization in the SI. Notably, although MHCII^+^Ly6C^low^CD64^+^ Mϕs constitutively produce large amounts of IL-10 compared to monocytes or DCs, IL-10 production did not change drastically during SI injury (Fig. 4c). These data suggest that constitutive IL-10 production, unlike inflammation-induced responsive IL-10 production, by MHCII^+^Ly6C^low^CD64^+^ Mϕs might play an important role in epithelial regeneration. However, we are unable to dissociate constitutive and responsive IL-10 production in this model, and hence it is difficult to conclude which type of IL-10 production (constitutive vs responsive) plays the more important role in epithelial regeneration.

The importance of IL-10-producing MHCII^+^Ly6C^low^CD64^+^ Mϕs in the regulation of intestinal homeostasis, particularly epithelial regeneration, has been described in different types of intestinal injury and/or inflammation. A recent report has shown that IL-10, produced by MHCII^+^Ly6C^low^CD64^+^ Mϕs, promotes epithelial re-generation in a colon biopsy–induced injury model^14^. In addition to murine models of intestinal inflammation and injury, IL-10-producing monocyte-derived Mϕs are important for the maintenance of intestinal homeostasis in humans. In the human intestinal mucosa, the HLA-DR^high^CD14^+^CD163^high^CD160^high^ Mϕ subset, which is thought to arise from peripheral blood CD14^+^ monocytes, has been identified as a significant source of IL-10 that is able to suppress the inappropriate proliferation of Th1/Th17 cells^21^. Interestingly, this subset is known to be diminished in patients with ulcerative colitis (UC)^21^. Since UC patients present with chronic mucosal ulceration, it is possible that the dysregulation of IL-10-producing intestinal Mϕs may lead to compromised epithelial regeneration in these patients.

The mechanisms by which IL-10-producing MHCII^+^Ly6C^low^CD64^+^ Mϕs promote re-epithelialization in the SI is unclear. In the colon biopsy–induced injury model^14^, IL-10 acts on colonic epithelial cells and activates CREB signaling and its downstream target WISP-1, which then promotes β-catenin/TCF signaling, epithelial cell proliferation and wound closure. Given this evidence, it is plausible that IL-10, produced by MHCII^+^Ly6C^low^CD64^+^ Mϕs, directly promotes the regeneration of epithelial cells in the SI. However, it remains possible that IL-10 primarily modulates immune cells, thus making its effect on epithelial healing indirect. In this regard, a recent report has demonstrated that IL-10 modulates CD11c^+^ myeloid cells in both the SI and colonic mucosa^22^. IL-10 signaling in CD11c^+^ mononuclear phagocytes, in turn, regulates T cell activation and the proliferation of intestinal crypt cells. Hence, the loss of IL-10 signaling in CD11c^+^ cells leads to augmented T cell activation as well as crypt hyperplasia^22^. Thus, IL-10, produced by the SI MHCII^+^Ly6C^low^CD64^+^ Mϕs, may also modulate other immune cells, including CD11c^+^ mononuclear phagocytes, and indirectly promote epithelial regeneration during SI injury.

Accumulating evidence has highlighted the importance of the gut resident microbes for epithelial regeneration. An earlier report has demonstrated that acute epithelial injury (dextran sulfate sodium (DSS)-induced colitis) in germ-free mice results in hypo-proliferation of colonic epithelial cells^12^. A similar defect is observed in mice deficient in Mϕs (op/op mice) and *Myd88*^12^. Additional studies have demonstrated that specific bacterial species or their metabolites elicit re-epithelialization in the colon. For example, *Akkermansia muciniphila* has been shown to be enriched in the proximity of damaged epithelium and promote wound healing in the colon^23^. Likewise, a microbial metabolite deoxycholate regulates the regeneration of colonic crypts, thus playing an important role in colonic epithelial repair^20^. The gut microbiota, in concert with intestinal macrophages, is clearly essential for epithelial regeneration. In this regard, activation of MyD88 signaling, elicited by the gut microbiota, is required for IL-10 production in colonic macrophages^10^. This notion is consistent with the importance of intestinal Mϕs and MyD88 signaling for epithelial recovery in the colon^12^. Unlike in the colon, we have demonstrated that the gut microbiota is dispensable for epithelial injury recovery in the SI. Depletion of the gut microbiota did not alter IL-10 production by SI Mϕs or the restoration of SI barrier function. This is consistent with our earlier finding that IL-10 production in the SI is independent of the gut microbiota and is instead regulated by dietary antigens^16^. It is noteworthy to mention that the role of the gut microbiota in SI injury remains controversial. Earlier studies suggested that the gut microbiota was crucial for the induction of NSAID-mediated epithelial injury in germ-free and gnotobiotic animals^24,25^. In contrast, more recent studies have demonstrated that the gut microbiota and its metabolites protect the host from SI injury and, therefore, depletion of the gut microbiota by antibiotics significantly exacerbates SI injury^26,27^. In our study, depletion of the gut microbiota did not affect susceptibility to SI injury. Heterogeneity of the gut microbiota composition across various animal facilities may account for these differences. Thus, further studies are needed to elucidate the impact of IL-10-producing Mϕs on epithelial recovery and its relationship with the gut microbiota. Another limitation is related to the background strain of mice used in this study. We used an inbred mouse strain (C57BL/6) in this study due to the availability of genetically engineered strains. However, immune responses and the resident microbiota may differ in outbred mouse strains. Further studies that use outbred mouse strains may provide more insight.

Dietary nutrients have been reported to modulate the development of regulatory immune cells and potentiate gut barrier function^28^. Since the abundance of some nutrients, particularly food-derived micromolecules, such as amino acid, is higher in the SI compared with the colon, the impact of dietary factors on the regulation of homeostasis should be more pronounced in the SI. In the context of Crohn’s disease, exclusive enteral nutrition (EEN) has been found to effectively induce remission with mucosal healing. Several clinical studies have shown this response to be particularly robust in the SI^29^. However, the precise mechanism by which EEN attenuates inflammation and improves Crohn’s disease symptoms remains largely unknown. Our previous study has demonstrated that IL-10 production by Mϕs in the SI is regulated by dietary amino acids^16^. Dietary amino acids activate the mTOR signaling pathway, which regulates the secretion of IL-10 from SI Mϕs through as yet unidentified mechanism^16^. Given this evidence, it is conceivable that amino acids included in EEN promote mucosal healing through the activation of IL-10 production by SI Mϕs.

Taken together, Mϕs and IL-10 produced by these cells largely contribute to the maintenance of epithelial barrier integrity in the SI. Since the development and function of anti-inflammatory Mϕs in the SI appear to be regulated by dietary factors, optimal dietary approaches could be used to promote mucosal healing in the SI, thus providing a more targeted option for a subset of Crohn’s disease patients.

## Methods

### Mice

Wild-type (WT) C57BL/6 and IL-10-deficient (*Il10*^−/−^) mice (C57BL/6 background), originally obtained from Jackson Laboratories (Bar Harbor, ME), were bred and kept under SPF conditions in the University of Michigan Animal Facility. IL-10 Venus reporter (*Il10*^Venus^) mice (C57BL/6 background) were generated as previously described^15^. CCR2 depleter (*CCR2*^DTR^) mice were kindly provided by Dr. Eric Pamer^30^. 8 to 16-week-old female and male mice were used in all experiments. SPF mice were fed a sterilized laboratory rodent diet 5L0D (LabDiet). All animal studies were performed in accordance with protocols approved by the Institutional Animal Care and Use Committee (IACUC) at the University of Michigan.

### Induction of indomethacin-induced small intestinal epithelial injuries

A single dose of indomethacin (IND) (15 mg kg^−1^, dissolved in 5% NaHCO_3_) (Sigma-Aldrich), injected subcutaneously^31^, was used to induce SI epithelial injury. Control mice were injected with the vehicle alone (5% NaHCO_3_). The following parameters were used to quantify the severity of SI injuries: body weight, survival rate, weight-to-length ratio of the SI, histology, cytokine mRNA levels in experimental tissues.

### Chemicals and Reagents

Diphtheria toxin (DT) was obtained from List Biological Laboratories (Campbell, CA. Cat. No. 150), reconstituted in PBS and stored at −80 °C. Mice received a dose of DT (10 ng g^−1^ in 0.1 ml PBS)^30^, injected intraperitoneal, every other day.

### Endoscopic examination of the murine small intestine

Mice were euthanized and SI tissues were harvested. Luminal contents were flushed with ice-cold PBS. A high-resolution (1,024 × 768 pixels), miniaturized endoscopic system (Colorview Veterinary Endoscope; Karl Stortz) was used to view the images of the SI mucosa^13^.

### Quantitative real-time PCR

Mucosal scrapings were collected from the proximal, middle and distal regions of the SI, and then placed in TRIzol reagent (Life Technologies). Purified RNA isolated from the tissues was reverse-transcribed into cDNA and then used for quantitative real-time PCR (qPCR) using SYBR Green Supermix (BioRad, Hercules, CA). The relative expression of target genes was calculated using β-actin as a reference gene. The following primer sets were used for amplifications: IL-1β-Fw; 5′-CAACCAACAAGTGATATTCTCCATG-3′, IL-1β-Rv; 5′-GATCCACACTCTCCAGCTGCA-3′, L-6-Fw; 5′-GAGGATACCACTCCCAACAGACC-3′, IL-6-Rv; 5′-AAGTGCATCATCGTTGTTCATACA-3′, TNF-Fw; 5′-GCCTCCCTCTCATCAGTTCT-3′, TNF-Rv; 5′-CACTTGGTGGTTTGCTACGA-3′, IL-10-Fw; 5′-CCCTTTGCTATGGTGTCCTT-3′, IL-10-Rv; 5′-TGGTTTCTCTTCCCAAGACC-3′, β-actin-Fw; 5′-AAGTGTGACGTTGACATCCG-3′, β-actin-Rv; 5′-GATCCACATCTGCTGGAAGG-3′.

### Intestinal permeabilization assay

Mice were denied access to food for 4 hrs prior to gavage with FITC-labeled dextran (500 mg kg^−1^, dissolved in PBS, MW 3000 – 5000; FD4, Sigma-Aldrich). Blood samples were collected 1 hour later and fluorescence intensity in serum was detected (excitation, 485 nm; emission, 525 nm; BioTek). FITC-dextran concentration was determined using a standard curve generated by serial dilution of FITC-dextran.

### Isolation of lamina propria mononuclear cells

Lamina propria mononuclear cells (LPMCs) were isolated from SI specimens using a modification of a previously described protocol^5,32^. Briefly, the dissected mucosal tissue was incubated in calcium and magnesium-free Hank’s balanced salt solution (HBSS) (Life Technologies, Carlsbad, CA) containing 1.5% heat-inactivated fetal bovine serum (FBS) (Life Technologies) and 1 mM dithiothreitol (Sigma-Aldrich) to remove mucus. Epithelial cells were removed by incubation in HBSS containing 1 mM EDTA (Quality Biological, Gaithersburg, MD) at 37 °C, repeated 2 times (first incubation; 10 min, second incubation; 30 min). The tissues were then collected and incubated, with agitation, in HBSS containing 400 U ml^−1^ of type 3 collagenase and 0.01 mg ml^−1^ of DNase I (Worthington Biochemical, Lakewood, NJ) for 90 min at 37 °C. The insoluble fraction was pelleted, re-suspended in a 40% Percoll solution (GE Healthcare Life Sciences, Pittsburgh, PA), layered on top of a 75% Percoll solution and centrifuged at 2,000 r.p.m. for 20 min at room temperature. Viable LPMCs were recovered from the discontinuous gradient interface.

### Flow cytometry analysis

Flow cytometry was performed using the LSR Fortessa (BD Bioscience) cytometer and analyzed using FlowJo software (Tree Star, Ashland, OR). Dead cells were excluded by 7-AAD staining. Non-specific antibody binding was blocked with an anti-CD16/32 antibody. Fluorescence-conjugated mAb against CD11b (M1/70), CD11c (N418), MHC class II (I-A/I-E) (M5/114.15.2), CD45 (30-F11), CD4 (GK1.5), CD3 (145-2C11), CD4 (GK1.5) and Ly6C (HK1.4) were from eBioscience. CD64 (X54-5/7.1) mAb was from BioLegend. Isotype-matched antibodies (eBioscience) were used for control staining. All antibodies were used at 1:100 dilution, except MHC class II, CD45 (used in 1:200 dilution). The concentration of cell suspensions was adjusted to 1 × 10^6^ cells per 100 μl.

### Statistical analysis

Statistical analyses were performed using GraphPad Prism software version 6.0 (GraphPad Software Inc.). Statistical tests used for experiments are provided in Figure legends. Differences of *P* < 0.05 were considered significant.

## Data Availability

The datasets generated during and/or analysed during the current study are available from the corresponding author on reasonable request.
